# Isolated Temporal Bone Metastasis With Inner Ear Invasion as Manifestation of Rectal Cancer

**DOI:** 10.7759/cureus.77501

**Published:** 2025-01-15

**Authors:** José A Ferreira, Paulo Luz, João F Simões, Filipa Simões

**Affiliations:** 1 Oncology, Unidade Local de Saúde (ULS) do Algarve, Faro, PRT; 2 Medical Science/Oncology, Universidade do Algarve, Faro, PRT; 3 Otolaryngology, Unidade Local de Saúde (ULS) do Algarve, Faro, PRT

**Keywords:** facial palsy, rectal cancer, temporal bone metastasis, temporal bone tumors, unilateral hearing loss

## Abstract

Temporal bone metastasis is a rare find. The most common sites of origin of temporal bone metastases are breast, lungs, and prostate. The main route of dissemination appears to be hematogenic. We present a case report of an 81-year-old male patient with locally advanced rectal cancer, without evidence of distant metastasis on CT of the chest, abdomen, and pelvis, presenting with rapidly progressive hearing loss, facial palsy, and loss of balance. Initial imaging is inconclusive, with meningioma being the leading hypothesis. However, histological analysis confirms the presence of carcinoma of colorectal origin, revealing an isolated temporal bone metastasis with inner ear invasion. The objective of this case report is to bring to attention that although metastasis of the temporal bone is rare, it should be considered in patients with otologic symptoms or facial palsy with a history of malignant tumors, even ones not usually associated with this metastasis location.

## Introduction

Temporal bone metastasis is a rare find, and rectal cancer represents a low percentage (one to three percent) of these malignancies [1,2]. The real incidence is unknown due to the rarity and misdiagnosis. Most frequently, these patients present with hearing loss, facial palsy, and otalgia. A considerable percentage appears to remain asymptomatic, which represents a diagnostic challenge [1-4]. The occurrence of these symptoms in patients with a history of malignancy should raise concern and prompt thorough investigation. This article presents a rare case of otogenic facial palsy and temporal bone metastasis originating from rectal cancer.

## Case presentation

An 81-year-old male patient presented to us with a diagnosis of locally advanced rectal cancer, without evidence of distant metastasis by computer tomography (CT) of the chest, abdomen, and pelvis. The only presenting symptoms were pelvic discomfort and diarrhea. The patient was proposed for neoadjuvant chemo-radiation therapy followed by surgery.

During the treatment planning, the patient started to exhibit a rapidly progressive unilateral mixed (conductive and sensorineural) right hearing loss, facial palsy (House-Brackmann scale IV), and vertigo. Initial examination showed no visible change in the external auditory canal or tympanic membrane at the time.

Initial cranial CT scan showed an expansive extra-axial temporal-petrosal lesion with adjacent bone sclerosis. After this finding, a high-resolution CT of the temporal bone and brain magnetic resonance imaging were decided. The exams were conducted 2 to 3 weeks after the initial CT, during which there had been worsening of symptoms, mainly vertigo and hearing loss, as well as developing of trismus.

**Figure 1:**
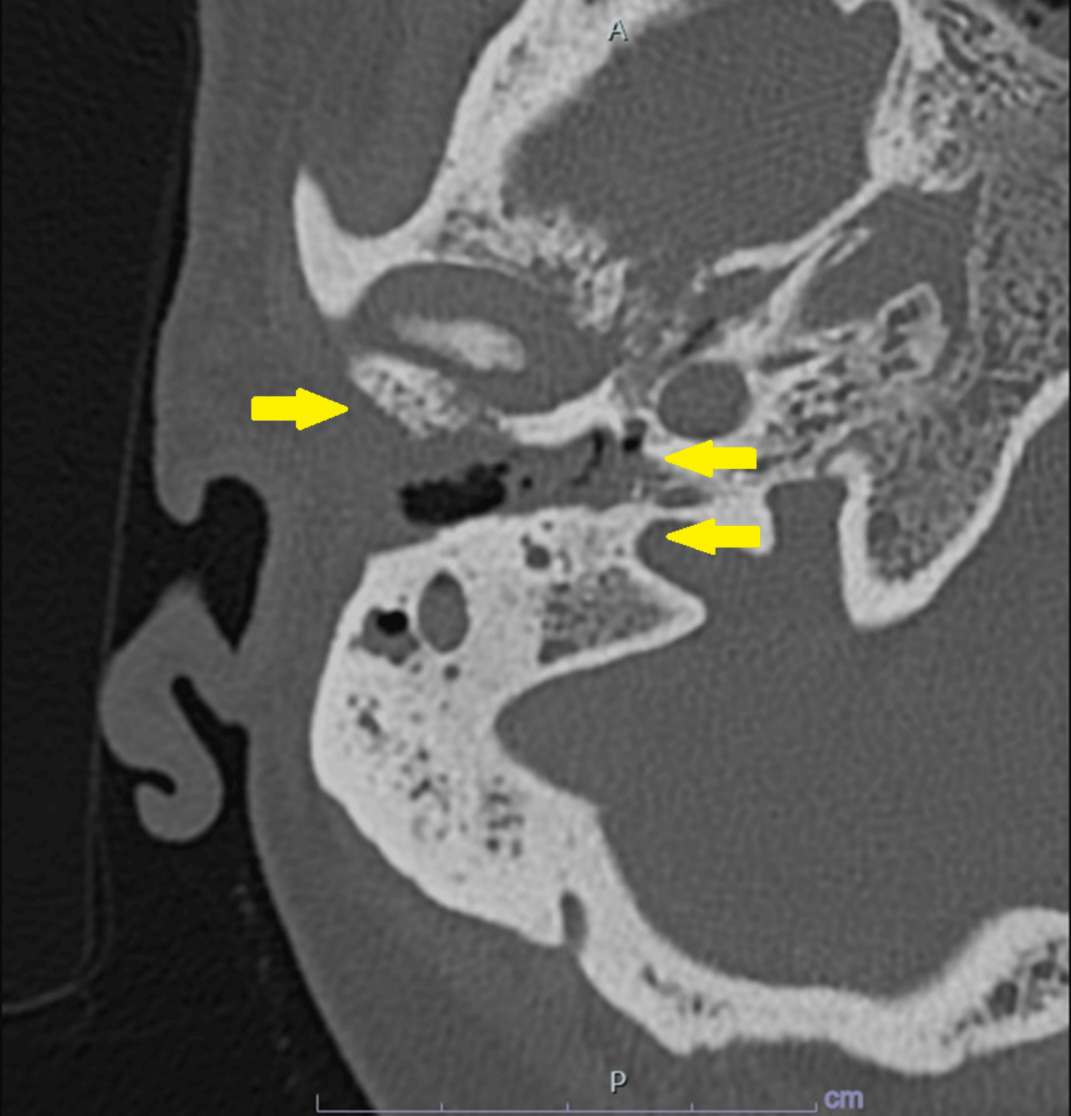
High-resolution CT scan of the temporal bone showcasing the osteolytic lesion, soft tissue invasion in the middle ear and mastoid.

**Figure 2:**
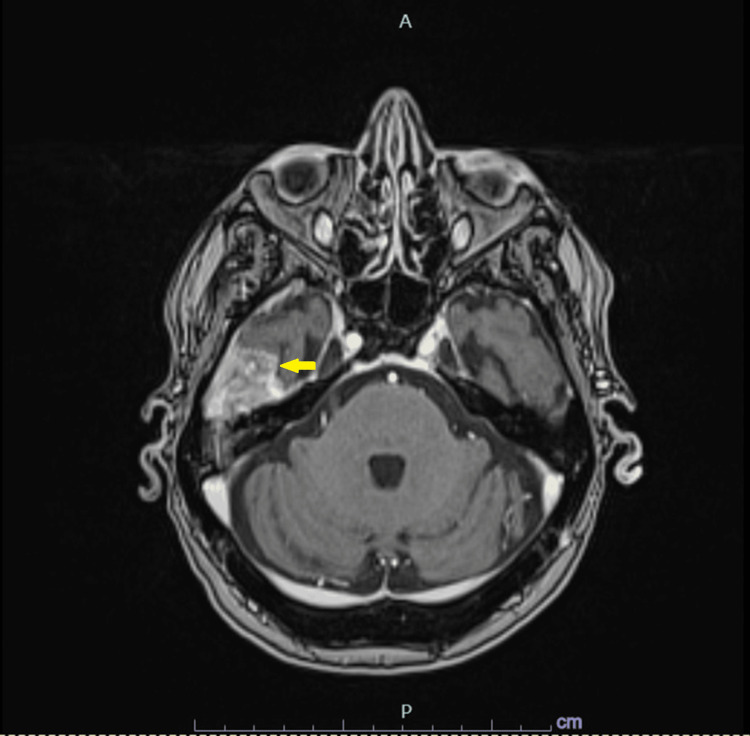
MRI of the brain showcasing the lesion with involvement of the middle fossa.

The CT and MRI results showed an osteolytic lesion with involvement of the glenoid cavity, middle fossa, and anterior wall of the auditory external canal, with a soft tissue expression to the inner ear (Figures 1, 2). Clinical examination at this time showed exophytic invasion in the outer 1/3 of the external auditory canal.

**Figure 3:**
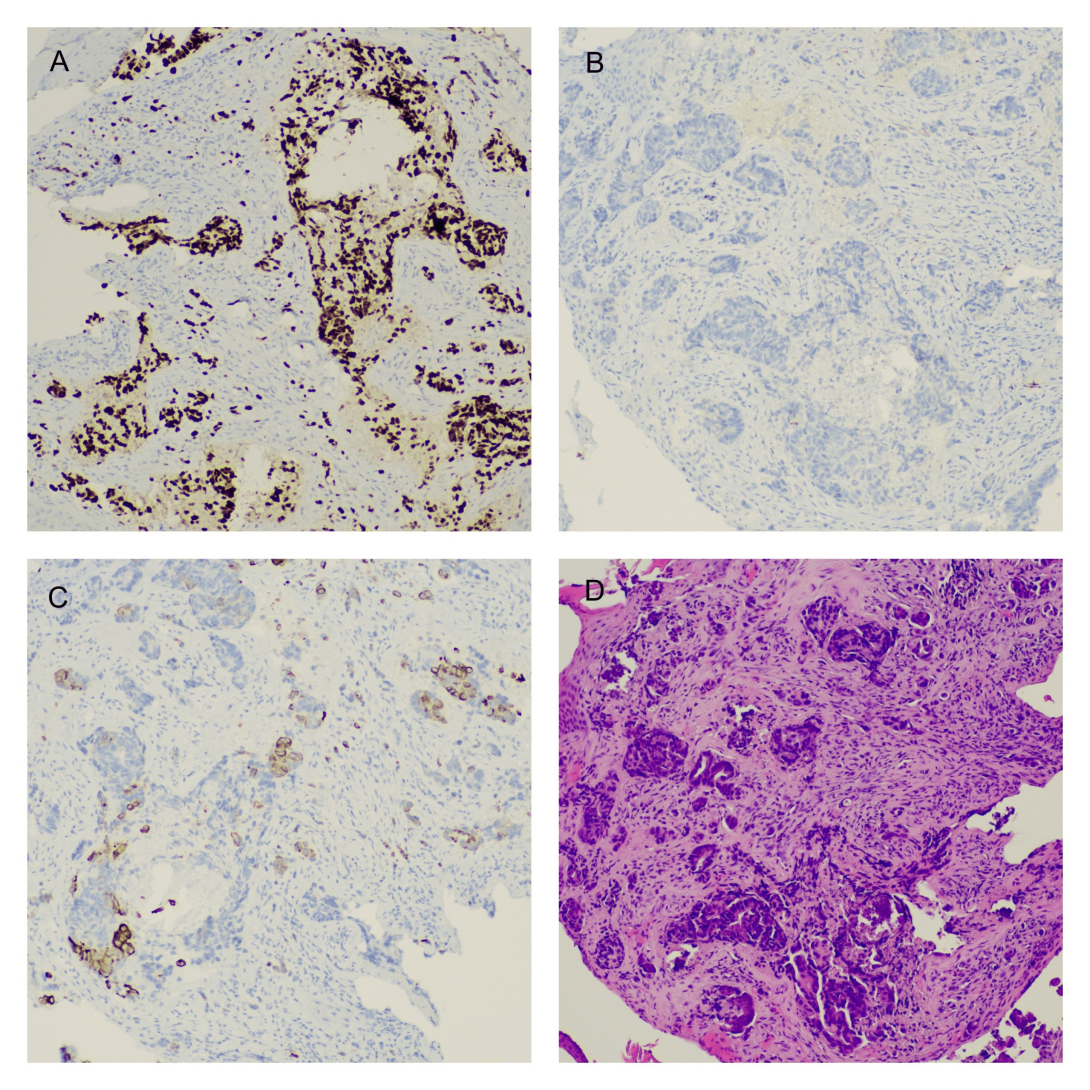
Histological microscopic images from the lesion biopsy. A. Immunohistochemistry stain for CDX2 at 100x magnification; B. Immunohistochemistry stain for CK7 at 100x magnification; C. Immunohistochemistry stain for CK20 at 100x magnification; D. Hematoxylin and Eosin stain at 100x magnification

However, these results were not sufficiently specific to determine the lesion's etiology, prompting the decision to perform a surgical biopsy from the external auditory canal. Histological analysis revealed invasive adenocarcinoma consistent with colorectal origin (CK20+, CDX2+, CK7-, TTF1-, PSA-) (Figure 3). Disease re-staging has been conducted at this time, which showed no evidence of other metastasis.

Shortly after the biopsy, the patient fell at his house, without evidence of any fracture, but with a severe compromise of his performance status. It was opted for a supportive care-only approach.

## Discussion

Colorectal cancer spreads lymphatic or hematogenous route, and the most common sites for metastization are the liver, peritoneum, and lungs. Bone metastases are rare, present in around three percent of metastatic colorectal cancer patients [5,6], and are usually associated with a poor prognosis [5].

Temporal bone metastasis is an even rarer find, with a few hundred cases reported for all primary tumors. The overall highest incidences for individual primary tumor sites included the breast (19.6%), lungs (16.1%), and prostate (8.6%). Rectal cancer represents a low percentage (one to three percent) of these malignancies. [1,2] The real incidence is unknown due to the rarity and misdiagnosis. Approximately 75% of metastasis to the temporal bone demonstrated hematogenous spread [2]. Most frequently, these patients present with hearing loss (44%), facial palsy (31%), and otalgia (17%). About one-third of patients remain asymptomatic [1-4].

Clinical examination of patients with temporal bone metastasis may show various findings, such as retroauricular soft tissue swelling or swelling of the tissue structures in the external auditory canal, inflammation (such as otitis media with effusion), and perforation of the tympanic membrane [7,8].

## Conclusions

Metastatic disease should always be considered in patients with facial nerve paralysis who have a history of malignancy. This case highlights the importance of recognizing that a seemingly localized condition, which could have been treated as such, presented with rapid, severe symptoms that might have easily been misdiagnosed as mastoiditis. It underscores the critical role of a thorough medical history and careful clinical reasoning in the evaluation of all patients.
